# Trends and Characteristics of Emergency Department Visits for Fall-Related Injuries in Older Adults, 2003–2010

**DOI:** 10.5811/westjem.2017.5.33615

**Published:** 2017-07-14

**Authors:** Kalpana N. Shankar, Shan W. Liu, David A. Ganz

**Affiliations:** * Boston Medical Center, Department of Emergency Medicine, Boston, Massachusetts; † Massachusetts General Hospital, Department of Emergency Medicine, Boston, Massachusetts; ‡ University of California, Los Angeles, David Geffen School of Medicine, Los Angeles, California; § VA Greater Los Angeles Healthcare System, Los Angeles, California

## Abstract

**Introduction:**

One third of older adults fall each year, and falls are costly to both the patient in terms of morbidity and mortality and to the health system. Given that falls are a preventable cause of injury, our objective was to understand the characteristics and trends of emergency department (ED) fall-related visits among older adults. We hypothesize that falls among older adults are increasing and examine potential factors associated with this rise, such as race, ethnicity, gender, insurance and geography.

**Methods:**

We conducted a secondary analysis of data from the National Hospital Ambulatory Medical Care Survey (NHAMCS) to determine fall trends over time by examining changes in ED visit rates for falls in the United States between 2003 and 2010, detailing differences by gender, sociodemographic characteristics and geographic region.

**Results:**

Between 2003 and 2010, the visit rate for falls and fall-related injuries among people age ≥ 65 increased from 60.4 (95% confidence interval [CI][51.9–68.8]) to 68.8 (95% CI [57.8–79.8]) per 1,000 population (p=0.03 for annual trend). Among subgroups, visits by patients aged 75–84 years increased from 56.2 to 82.1 per 1,000 (P <.01), visits by women increased from 67.4 to 81.3 (p = 0.04), visits by non-Hispanic Whites increased from 63.1 to 73.4 (p < 0.01), and visits in the South increased from 54.4 to 71.1 (p=0.03).

**Conclusion:**

ED visit rates for falls are increasing over time. There is a national movement to increase falls awareness and prevention. EDs are in a unique position to engage patients on future fall prevention and should consider ways they can also partake in such initiatives in a manner that is feasible and appropriate for the ED setting.

## INTRODUCTION

Falls among older adults (those at least 65 years of age) are frequent with approximately a third of community-dwelling older adults falling each year.^1^ The estimated annual direct medical cost of non-fatal fall-related injuries is approximately $31.3 billion and will increase in the future as the population ages.^2^ Not only are falls frequent and costly, they are the number one cause of unintentional injury leading to death among the elderly.^3^ There are more than 10,000 deaths and 2.6 million nonfatal injuries from falls among older adults annually.^4^ Approximately 10% of falls result in significant injury.^5^ Falls increase the risk of admission to nursing homes^6^ and future falls,^7^,^8^ and are associated with health decline, social isolation and loss of confidence.^9^–^11^

In 2006, older adults made more than two million visits to the emergency department (ED) for injurious falls, representing 10% of ED visits among this group.^12^ Over two thirds (70.4%) of these patients were discharged after their ED visit, with the remaining 29.6% admitted to the hospital.^12^ Annual estimated costs of ED visits for falls is $8.5 billion.^2^ Given that falls are a potentially preventable cause of injury, functional decline and traumatic death,^13^–^15^ EDs are in a unique position to evaluate and potentially intervene on behalf of these patients.

Since the number of fall-related emergencies is likely to rise as the population ages, it is important to understand the characteristics and trends of ED fall-related visits among older adults. To date, we are unaware of studies evaluating ED visits in the United States across time for fall-related complaints among the elderly. Our objective was to determine fall trends over time by examining changes in ED visit rates for falls in the U.S. between 2003 and 2010, detailing differences by gender, sociodemographic characteristics and geographic region. We hypothesized that falls among older adults are increasing and examined potential factors associated with this rise, such as race, ethnicity, gender, insurance and geography.

## METHODS

### Study Design and Setting

We conducted a secondary analysis of data from the National Hospital Ambulatory Medical Care Survey (NHAMCS), publicly available through the Centers for Disease Control and Prevention (CDC). The NHAMCS is a national probability-sample survey of patient visits to selected ambulatory care departments conducted annually since 1992 by the CDC’s National Center for Health Statistics (NCHS). For this analysis, we included data solely from the ED visit files of calendar years 2003- 2010 during which a purposeful sample of 386 to 443 EDs were included. Each patient visit was weighted to form national estimates for all components of the survey.^16^ The resulting overall, unweighted response weights ranged from 82.5% to 89.2%.

Our subpopulation of interest was patients aged 65 or older whose ED visit was related to a fall. We contacted NCHS to identify the International Classification of Diseases, Ninth Revision (ICD-9) external cause of injury codes used to classify a fall, where each visit can list up to three causes of injury. A variable was created to classify all fall-related visits from the cause of injury variables (using ICD-9 external cause of injury codes 880.0–888.9). Any fall-related causes listed in the three-causes-of-injury data fields were classified as “fall.” We stratified all visits to EDs during this time period by age, sex, race, ethnicity, insurance status and region.

The total number of unweighted patient visits from years 2003–2010 among those age 65 or older was 42,089, and the total number of unweighted patient visits among those age 65 or older with a fall-related visit from years 2003–2010 was 5,512. Although we focused only on the subpopulation of those age 65 or older, all observations remained in the analyses in order to correctly calculate the estimates.

Population Health Research Capsule

What do we already know about this issue?

Older adult falls are costly to both the patient in terms of morbidity and mortality and to the health system, but are a preventable cause of injury.

What was the research question?

To determine fall trends over time by examining changes in ED visit rates for falls in the United States between 2003 and 2010.

What was the major finding of the study?

The overall visit rate for fall-related injuries among people age ≥ 65 increased from 60.4 to 68.8 per 1,000 population (p=0.03).

How does this improve population health?

There is a national movement to increase falls awareness and prevention. EDs are in a unique position to engage patients on future fall prevention in ways that are feasible in an ED setting.

We managed and analyzed all data using SAS 9.4 (SAS Institute Inc., Cary, NC) and STATA/IC 13.1. Because we used a publicly available dataset, this study was deemed exempt from review.

### Statistical Analysis

**We analyzed** all data using the sampled visit weights, which account for the specific sampling design of NHAMCS; unweighted numbers were not used to calculate estimates. For the subpopulation age 65 and older, we calculated rates for fall-related ED visits by age, gender. race, region, and source of payment. Rates were calculated for each year from 2003–2010 as the number of weighted visits per 1,000 population. We obtained population data from the U.S. Census Bureau for each rate calculated, depending on the specific subpopulation. For each subgroup, a special weight variable was created using the appropriate population estimate as the denominator. We used SAS survey procedures with the appropriate “cluster” and “strata” design variables to account for the complex nature of the sample; weighted frequencies and 95% confidence limits were calculated. All visit rates were calculated per 1,000 population. To ensure reliability of estimates reported, we did not include rates if unweighted sample sizes were less than 30.

We used simple linear regression models to assess trends in rates across years 2003–2010. For each model, year was used as the dummy variable and the respective population rate as the dependent variable. We calculated rate differences (RD) over the seven-year period (2003–2010) using a linear regression model to assess the annualized rate change per year, measured as a continuous variable. This is represented as an annual change per 1,000 persons, with significance assessed at the p<0.05 level. No adjustments for multiple comparisons were made since the analyses were exploratory in nature.

## RESULTS

**Figure 1:**
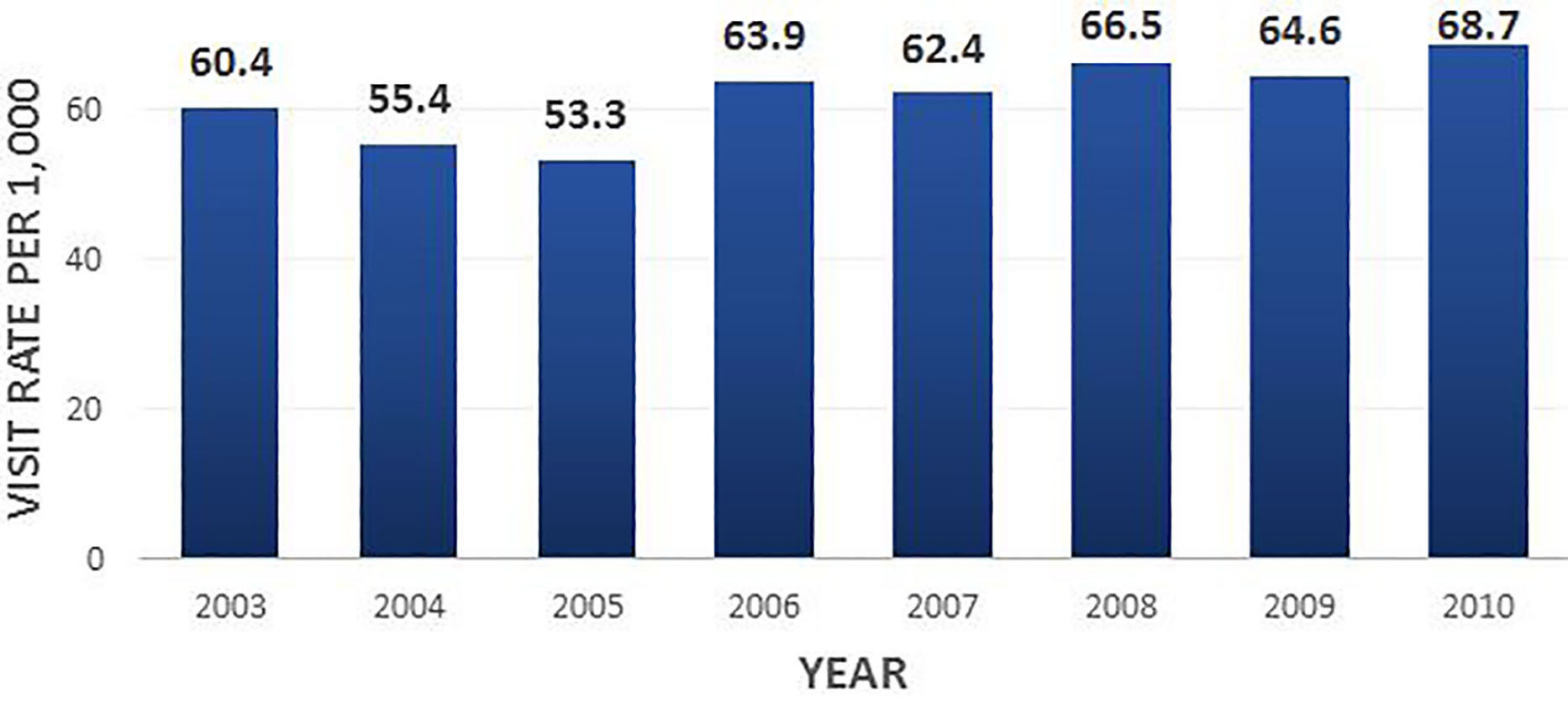
Fall-related ED visit rates by year, for patients 65 and older, 2003–2010.

**Figure 2:**
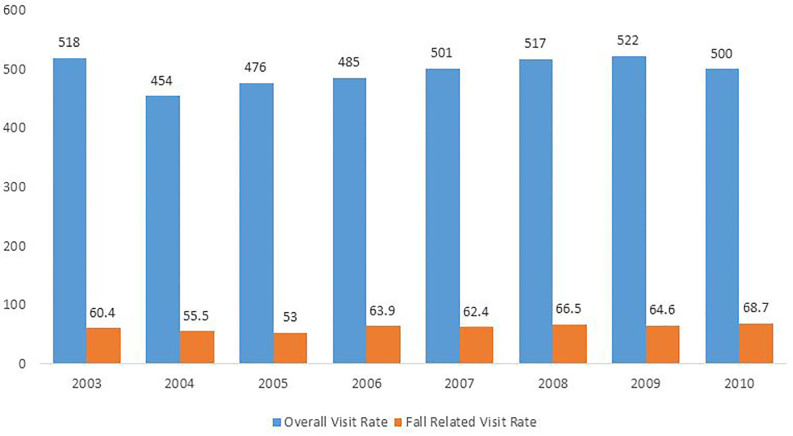
Estimated fall-related ED visits per 1,000 compared to overall ED visits per 1,000 (65 and older)

We found that ED visits for falls in adults 65 years and older increased over the seven-year period by 27%, ranging from 2.2 to 2.8 million visits. Between 2003 and 2010, the visit rate for falls and fall-related injuries increased from 60.4 (95% confidence interval [CI] [51.9–68.8]) to 68.8 (95% CI [57.8–79.8]) per 1,000 population; on an adjusted basis, there was an annual visit rate increase of 2.3 per 1,000 (p= 0.03) (Table 1, Figure 1). There was also an increase in the overall visit rate for this population group over time (Figure 2).

Controlling for U.S. population growth, visits rates for falls continued to grow. Specifically, visits by patients age 75–84 years accounted for the greatest rate increase with rates increasing from 56.2 to 82.1 per 1,000 population age 65 and older (annualized RD 4.5 per 1,000, 95% CI [1.8–7.3], p <.01), while visit rates for patients 65–74 years and 85 years and older remained unchanged (Table 1). ED visits by women increased from 67.4 to 81.3 (RD 3.5, 95% CI [0.1–6.9], p = 0.04) while the ED visit rate by men did not change significantly over time. There was also an increased rate of non-Hispanic Whites visiting the ED over time for falls from 63.1 to 73.4 (RD 3.4, 95% CI [0.5–6.4], p < 0.01). By region, older adults from the South had the highest increase in the rate of people who fell, from 54.4 to 71.1 (RD 5.3, 95% CI [0.6–10.2], p=0.03), but overall the Northeast had the highest rate of fallers, ranging from as low as 56.2 to as high as 91.8. ED visit rates among adults with Medicare as their primary insurance also significantly increased in this time period from 51.4 to 65 (RD 4.5, 95% CI [0.5–8.]7, p= 0.03). Patients on Medicaid had higher rates of falls from 2005 to 2007, but visual inspection of the data over the entire 2003–2010 timespan (Table 2) did not reveal a consistent pattern. Visit rates remained unchanged for those with private insurance, and declined for those who were uninsured or had other types of payment methods as their primary insurance (RD −8.0, 95% CI [−14.4- −1.2], p<0.01).

The year 2005 is documented to have the lowest number of total visits for falls with a visit rate of 53 (95% CI [45.6–60.3]), driven by a nadir in the visit rate for adults 85 and older over the seven-year period (Table 2).

## DISCUSSION

Between 2003 and 2010, the total annual visits to U.S. EDs for a fall or fall-related injury increased over time by 27% over the seven-year period. This trend was particularly pronounced among patients between the ages of 75–84, female patients, non-Hispanic Whites and patients residing in the South. Compared to existing regional and state-based data on fall trends, our study examines national fall trends over a longer time span and with a larger cohort and also identifies a variety of epidemiological factors that may contribute to this rising number.

One reason for increasing ED fall visits over time may be due to all ED visits increasing in this population despite improvements in primary care access^17^ (Figure 2). A recent report released by the American Hospital Association examining trends in ED use by Medicare beneficiaries between 2006 to 2010 showed a number of factors contributing to this, including rising severity of illness of beneficiaries receiving ED care, greater use of ED services by people dually eligible for Medicare and Medicaid who are generally sicker with multiple chronic conditions, and increasing use of ED services by beneficiaries with behavioral health diagnoses who require higher intensity of services.^18^ While the number of primary care clinics accepting Medicare remains strong, there is recent evidence to suggest that practices accepting new Medicare patients are dwindling,^19^ with many patients still unable to access clinics after business hours.^20^ A combination of these factors is likely contributing to the overall increasing ED visit rates for falls as well.

We found that ED visits for falls are particularly increasing among patients between the ages of 75 to 84, after controlling for population growth. Falls are events driven by multiple interacting causes. One explanation for increasing ED visits may be an increase in frailty and disability among older people living at home or in nursing homes. Based on recent population data, life expectancy has increased since 2000,^21^ particularly among White males, while death rates for cardiovascular and pulmonary disease have decreased among patients 65 years and older compared to the 1990s. However, death rates from unintentional injuries such as falls have increased over time for this age bracket.^22^ If improved medical care and interventions help people to live longer with diseases that historically would have caused them to die, then more people are living with underlying comorbidities contributing to their overall frailty and fall risk. There is one study demonstrating an increase in frailty and disability of patients living at home over time, but this was based on self-report.^23^

We found an increase in ED fall visits by women over our study period. In contrast, it does not appear that the ED visit rate of male fallers has changed over time. This could be due to a number of reasons. First, women tend to live longer than men. This phenomenon has not changed over time and may be reflected in a larger numerator or in the continued increased willingness of women to go to an ED to seek care than men. It is also possible that men may come to the ED with more detrimental injuries from a fall and only present with one serious injury-related visit versus women who tend to suffer recurrent falls.^4^,^24^ There are data suggesting men are more likely to die from a fall, possibly because they suffer from more comorbid conditions than women of the same age or they are potentially partaking in riskier activities such as climbing ladders, which is not changing over time.^25^ Lastly, it is possible that men are seeking emergency care for injuries but not endorsing or being coded for a fall.

The finding that non-Hispanic Whites are at a higher risk of falling has been documented in prior studies and this predisposition does not appear to have changed over time. 26–28 The literature demonstrating the surface upon which patients land also differs with Black individuals landing on more indoor-type and non-Hispanic White individuals tending to land on outdoor-type surfaces.^29^ If riskier activities involving walking while hurrying, working in the yard or garden, or carrying something bulky impart a higher overall likelihood of falling,^30^ it is possible that non-Hispanic Whites have fewer mobility issues to allow them to partake in more outdoor, risk-taking behaviors, which contributes to their higher rate of falls. What is unclear is why there is a trend towards increasing rates of ED visits for this group as compared to other races. It is possible this is due to an increase in any given fall risk factor, such as heart disease, medications, an increase in risky behavior, decreased ED access for minorities or limited uptake of fall prevention programs, as described above. Due to the serial cross-sectional nature of this data, interpretations are limited and the findings are not controlled for other factors.^31^,^32^ Further studies are needed to assess longitudinally what factors are driving this finding.

Older adult patients residing in the South are also increasingly visiting EDs for falls. As falls are strongly associated with fractures, especially among osteoporotic patients, our findings are consistent with data indicating that fractures of the hip, spine and extremity are also higher in the South. One explanation may be from intrinsic patient factors that are increasing a patient’s risk for a fall.^33^–^37^ Lauderdale et al. studied regional variations for hip fractures and found that patients who grew up primarily in the South had an increased risk of fractures versus patients who only resided in the South in their older years. The author postulates that determinants present at a younger age, such as lifestyle or poor nutrition in the southern region, are driving this overall risk.^38^ These determinants may also be contributing to the higher risk of falls in the South over time; however, studies are needed to further elucidate this.

A second explanation may be due to extrinsic factors beyond the patient’s control. There is evidence to suggest that poorer socioeconomic status is associated with a higher risk of falls in part due to poor housing, roads and sidewalks and surrounding environments.^39^–^41^ Based on U.S. Census data the South has had the highest percentage of poverty as compared to the rest of the country since 1950,^42^ which may be contributing to the increasing rate of falls in this area; however, further research is needed to assess this association and understand if other factors are mediating this effect.

Interestingly, it appears that there is an increasing rate of falls despite national falls-prevention initiatives. Many of these initiatives involve linking to community falls programs and incorporating screening algorithms into office-based practice. Such initiatives are challenging to implement due to their cost, time requirements, need for adaptation and limited use by the community. Despite the potential effectiveness of fall-prevention programs, participation ranges from 15% to 50% with women having higher enrollment and completion rates than men.^43^,^44^ With such low participation rates, it appears that such barriers are not easily resolved and their positive effects over time may not be captured during the time frame of this data.^45^,^46^

Despite low participation it is clear that EDs have a unique window of opportunity to educate these patients on the morbidity and mortality associated with falls while they are still being treated for their fall-related injury, as well as motivate ED providers to collaborate with primary care and community-based organizations to reduce future falls. Such interventions may include involving physical or occupational therapists in the ED to evaluate, educate and potentially introduce use of assistive devices such as walkers or canes, providing handouts or showing short videos, referring to a dedicated falls clinic and engaging with community partners who run evidence-based balance and strength classes for fall prevention.

## LIMITATIONS

Our study had a number of limitations. First, the NHAMCS surveys use the U.S. Census Bureau as the field data collection agent, which can introduce error into the dataset. We were specifically concerned with the falls rate increase from 2005 to 2006 and the reported data on Medicaid. This issue was somewhat mitigated through completeness checks on receipt of the data by NHAMCS itself. In terms of the falls rate from 2005 and 2006, we specifically asked the CDC and evaluated the data collection tools spanning across all the years to assess whether the falls documentation changed over time. The last reported changes in the way injuries were coded were in 1997 and confirmed through phone conversations with the CDC and NHAMCS specialists and thus do not offer a clear explanation for this finding. The Medicaid data are difficult to interpret due to the wide confidence intervals, suggesting a small sample size. We report these data for the sake of completeness but acknowledge we cannot make any statements regarding the size or the trends of this number.

Second, NHAMCS surveys themselves may include inaccuracies in the data fields as the responses are self-reported; however, there is low probability of differential misreporting over time to bias our results. Third, as NHAMCS data are cross-sectional, we do not know if new patients are frequenting the ED for falls or the increased trend is derived from individual patients presenting with repeat falls. Previous studies have demonstrated an 18% recidivism^47^ rate within one year, which may account for our numbers; however, we would expect to see this reflected across all the years, which would not account for the overall upward trend. We also do not know how the trend in use of EDs for falls relates to a shift away from office practices for fall-related visits or if patients are sustaining more injurious falls over time, which would account for an upward trending ED visit rate. Fourth, we analyzed available data from 2003 to 2010. Since the initial analysis began, two more years’ worth of data has been made available and would be worthwhile for future studies to reassess these trends.

## CONCLUSION

Our findings suggest that over time, older adults are presenting to the ED with falls at an increasing rate. While many of the characteristics we examined cannot be changed, ED fall patients can be risk stratified to prevent subsequent falls. EDs are generally involved with the treatment of the acute injury as a result of the fall but are infrequently involved in any prevention activities or referrals, especially if these older adults are discharged back to the community. As older-adult falls are becoming a more widely discussed public health issue through various policies, the CDC’s recent development of the Stopping Elderly Accidents, Deaths and Injuries (STEADI) toolkits^48^ and a large national movement for fall prevention,^49^ EDs have a potential opportunity to engage in future fall-prevention interventions given their fall visit volume and unique teachable moments. Further research should assess what types of interventions are appropriate and feasible to be initiated in the ED setting.

## Figures and Tables

**Table 1:** Fall-related emergency department visits in the United States among ages 65 and older, 2003–2010.

	ED visits, unweighted no.		Estimated ED visits, weighted no. in millions		Estimated ED visits per 1000 no. (95% CI)	p value for linear trend*
		
	2003	2010	2003	2010	Annualized Rate Difference per 1,000 over time	
Total visits (fall)	799	722	2.2	2.8	2.3 (0.3, 5.4)	0.03
Age (years)						
65–74	257	213	0.7	0.9	2.1 (−1.5, 5.9)	0.20
75–84	285	289	0.7	1.1	4.5 (1.8, 7.3)	< 0.01
85 and older	257	220	0.8	0.8	1.9 (−4.0,8.3)	0.46
Gender						
Male	266	237	0.8	0.9	1.6 (−1.8, 5.2)	0.29
Female	533	485	1.4	1.9	3.5 (0.1, 6.9)	0.04
Race/ethnicity						
White (non-Hispanic)	678	596	1.9	2.4	3.4 (0.5, 6.4)	<0.01
Black	56	54	0.2	0.2	1.1 (−5.1, 7.6)	0.69
Hispanic or Latino	42	43	0.1	0.2	1.2 (−6.4, 9.4)	0.72
Other race	29	33	0.05	0.08	−6.6 (−15.0, 2.7)	0.10
Region						
Northeast	218	199	0.6	0.6	0.2 (−4.9, 5.5)	0.94
Midwest	184	147	0.5	0.6	1.8 (−2.4, 6.2)	0.35
South	216	239	0.7	1.1	5.3 (0.6, 10.2)	0.03
West	181	137	0.4	0.6	3.2 (−0.3, 6.8)	0.07
Primary source of payment						
Medicare	609	616	0.6	0.6	4.5 (0.5, 8.7)	0.03
Medicaid	27	17	0.03	0.02	−7.3 (−36.0, 34.4)	0.63
Private insurance	114	54	0.1	0.05	−1.8 (−8.5, 5.5)	0.56
Self-pay, other or unknown	49	35	0.05	0.03	−8.0 (−14.4, −1.2)	0.03

*CI*, confidence interval; *ED*, emergency department.

* P-value based on the linear regression trend from 2003 and 2010

**Table 2:** Fall-related emergency department visits per 1,000 population in the United States among ages 65 and older, 2003–2010. RATES FOR EACH YEAR, 2003–2010

	Estimated ED visits per 1,000 population 65+, no. (95% CI)							
	
	2003	2004	2005	2006	2007	2008	2009	2010
Total visits (fall)	60.4 (51.9, 68.8)	55.4 (47.6, 63.1)	53 (45.6, 60.3)	63.9 (53.4, 74.3)	62.4 (53.2, 71.6)	66.5 (55.7, 77.4)	64.6 (54, 75.2)	68.7 (57.7, 79.7)
Age (years)								
65–74	36.5 (29.3, 43.8)	33.4 (27.4, 39.5)	32.6 (26.5,38.8)	39.6 (28.9, 50.3)	32.9 (26.2, 39.7)	33.7 (25.2, 42.1)	42.7 (33.3, 52.1)	39.8 (29.9, 49.6)
75–84	56.2 (46.1, 66.2)	63 (51.9, 74.2)	61.9 (50.8, 72.9)	67.6 (53.7, 81.5)	65.8 (53, 78.5)	76.2 (63.2, 89.3)	64.7 (51.3, 78)	82.1 (67.1, 97.1)
85 and older	168.1 (133.3, 202.9)	120.4 (96.5, 144.3)	106.8 (82.9, 130.7)	144.9 (115.5, 174.2)	162.5 (134.2, 190.9)	164.5 (130.5, 198.6)	145.2 (110.5, 179.9)	151.4 (124.2, 178.6)
Gender								
Male	50.5 (40, 61)	39.1 (31.4, 46.8)	47.8 (39.2, 56.5)	43.6 (35.5, 51.8)	44.7 (35.8, 53.6)	48.9 (39.1, 58.7)	47.4 (37.8, 57.1)	52.2 (41.8, 62.6)
Female	67.4 (57.8, 76.9)	67 (57, 76.9)	57.6 (48.3, 66.9)	78.5 (64.2, 92.8)	75.3 (63, 87.6)	79.5 (64, 95.1)	77.2 (62.3, 92.2)	81.3 (66.7, 95.8)
Race/ethnicity								
White (non-Hispanic)	63.1 (53.6, 72.6)	55 (46.5, 63.4)	55.3 (46.9, 63.6)	66.1 (54.4, 77.7)	63.9 (54.1, 73.7)	70.5 (58.2, 82.7)	68.3 (56, 80.6)	73.4 (60.4, 86.3)
Black	56.7 (34.6, 78.9)	60.5 (42.1, 78.8)	53.6 (31.3, 76)	56.6 (28.3, 84.9)	79.8 (48, 111.6)	47.2 (27.1, 67.2)	69.4 (43.5, 95.3)	56.9 (29.9, 83.9)
Hispanic or Latino	54.3 (31, 77.6)	61.9 (40.7, 83.2)	44.4 (23.5, 65.3)	55.3 (32.2, 78.4)	46.7 (24.9, 68.5)	75.1 (45.4, 104.8)	38.7 (20.8, 56.6)	59.3 (30.4, 88.1)
Other race	51.5 (20.7, 82.2)	83.1 (35.5, 130.7)	46.2 (3.5, 88.9)	74.3 (33.6, 115)	48.4 (23.5, 73.3)	60.1 (30.9, 89.2)	40.9 (16.2, 65.6)	41.2 (13.7, 68.7)
Region								
Northeast	75.3 (57.8, 92.8)	73 (56.4, 89.5)	58.5 (40.9, 76.1)	74.2 (58.8, 89.7)	56.2 (40.4, 71.9)	91.8 (59.9, 123.7)	64.7 (49, 80.4)	77.1 (60.2, 94)
Midwest	60 (43.4, 76.6)	60.1 (43.2, 77)	60.3 (46.6, 74.1)	60.5 (39.4, 81.7)	75.6 (56.1, 95)	55.1 (34.3, 76)	70.2 (42.3, 98)	62.7 (37.3, 88)
South	54.4 (40.7, 68.1)	43.7 (32.1, 55.4)	45.8 (32.8, 58.9)	65.1 (44.1, 86.1)	62.1 (47.3, 76.9)	57.3 (43.1, 71.5)	65.1 (46.7, 83.5)	71 (50.8, 91.2)
West	56.2 (34.5, 77.9)	52.9 (33.2, 72.6)	51.9 (37, 66.7)	55.3 (34.5, 76.1)	54.5 (30, 79)	71.4 (45.5, 97.2)	57.5 (37.7, 77.4)	65.3 (42.7, 87.8)

*CI*, confidence interval; *ED*, emergency department.
